# Anatomical, Pathological, and Therapeutical Researches on the Disease Known by the Names of Typhoid Fever, &c.

**Published:** 1841-07

**Authors:** 


### Art. II.

*Recherches Anatomiques, Pathologiques et Thérapeutiques, sur la Maladie connue sous les noms de Fièvre Typhoïde, Putride, Adynamique, Ataxique, Bilieuse, Muqueuse, Gastro-entérite, Entérite Folliculeuse, Dothinentérie, &c. comparée avec les Maladies aigues les plus ordinaires.* Par P. C. A. Louis, Médecin de l'Hôtel-Dieu, Président perpétuel de la Société Médicale d'Observation de Paris, &c.—*Paris,* 1841. Deuxième Edition, 2 tomes 8vo, pp. 542, 523.

*Anatomical, Pathological, and Therapeutical Researches on the Disease known by the names of Typhoid Fever,* &c. By P. C. A. Louis, Physician to the Hôtel-Dieu, perpetual President of the Medical Society of Observation, &c.—*Paris,* 1841.

Few works have exercised a more important influence on the medical science of their age than that before us, whether we consider it as contributing to dethrone the long reigning (but at the time of its appearance already tottering) dogma of the essentiality of *all* fevers, or as demonstrating the fallacy of the seductive and grasping doctrine of Broussais, or as containing the earliest exhibition of the practical workings of the system of studying the science devised by its gifted author: probably its chiefest merit, because that ensuring the largest amount of practical good, arises out of the characteristic last referred to. Before the production of these volumes it might, on the evidence of the previous history of our science, have been justly doubted whether any pathologist might arise capable of acting uniformly and exclusively on the principle, in the recognition of which the successful cultivation of all sciences of observation is involved, enunciated in the following words: "je sais que la vérité est dans les choses et non dans mon esprit qui les juge; et que moins je mets du mien dans les jugemens que j'en porte, plus je suis sûr d'approcher de la vérité." It was not to be doubted that one day some keen spirit must speculatively admit the applicability of Rousseau's apophthegm to the study of medicine, and recognize its dependence upon the philosophy of Bacon; that this had actually been done, though imperfectly, by others is even strictly true. But to announce and profess the notion that any medical doctrine having pretensions to an origin in sound philosophy, must of necessity be inductive and purely inductive in its nature, was one thing;—to demonstrate the truth of this by practical evidence another. To work upon such notion to the exclusion of all others for years, to overcome the almost unconquerable tendency of the mind to speculate and not to *induce*, to bestow lengthened toil in the discovery of certain results, while others of equal apparent value might, on the old and legitimised system, have been quasi-established by a quiet exercise of the simplest thought, required a degree of exalted zeal, of perfect mental self-control and of enlightened love of practical truth for its own sake, which the tendencies of the past cultivators of our science gave us no right to expect in their successors.

And the present appears an exceedingly appropriate time for the introduction of this work to the British public. There never was in truth a period when all questions relating to the nature, modes of origin and propagation of fever, excited more attention among us than the present, or when conscientious men more eagerly desired an accurate examination, based upon facts and—as far as this is possible—upon facts *alone*, of the great question of the identity or dissimilarity of the fièvre typhoïde of Paris and the typhus fever of this country. To institute this examination as effectively as we can with the *positive* materials within our reach will, we believe, be to render a service to all interested in the philosophic settlement of such questions. As the essential and necessary basis of the enquiry, we shall set out with an analysis of the work before us, as complete and circumstantial as the nature of this journal will justify. A mere outline of its contents would not suffice for our purpose. Though it is, we are quite aware, very commonly supposed that the leading facts established by M. Louis are at the present day accurately understood among us, the truth is, on the contrary, that evidences of individual ignorance of the essential substance of his doctrine are daily made public; and still, as of old, the idea of acquiring acquaintance with details is, practically speaking, heartily scoffed at.

In addition to the distinction between this and other *ex professo* treatises on fever afforded by its strictly inductive character, there is another peculiarity in its construction, which at once exhibits the comprehensiveness of the views taken of disease by its author, and adds vast weight to—or, more correctly speaking, creates the importance of—the general deductions drawn by him in respect of typhoid fever from his different series of particular facts. It is this. Recognizing the important truth, that in order to establish the real value of any symptom or set of symptoms in connexion with any given disease, it is necessary to ascertain not only the degree of frequency with which it or they may attend that disease, but also the degree to which they are—either singly or in combination, in the same or in a different order—peculiar or proper thereto, he conceived the series of elaborate and minute comparisons instituted in these volumes, on the occasion of each symptom and lesion, between typhoid fever on the one hand, and all other acute diseases collectively on the other. The work thus supplies an analysis of nearly nine hundred cases of acute disease, and should rather be entitled a treatise upon the philosophical pathology of acute disease generally, and of typhoid fever in particular, than a simple monograph of the latter affection.

The work is divided into four parts. In the First of these are reported the histories of eighteen subjects whose death occurred at different periods of typhoid fever, exhibiting the varieties in the condition of the parts implicated, dependent on the greater or less duration of the malady. The Second contains a general description of the anatomical changes observed in all the fatal cases both of typhoid fever and of other acute diseases,—an exposition of the causes of death in both series of cases, and a brief summary of all the morbid alterations described. In the Third part, the symptoms in the fatal cases and in those terminating by recovery of the patient are analyzed; the subjects of "latent" and of "simulated" typhoid fever are examined; the epiphenomenon of intestinal perforation described; and the causes of the disease enquired into. The Fourth division embraces the subject of treatment. Under these different heads some new matter appears in the present edition. The anatomical chapters are enriched with observations on the work of M. Chomel, with contributions from M. Barth, and a minute description, furnished by MM. Rilliet and Taupin, of the lesions discovered in the bodies of children, (from ætat. two to fifteen, both inclusive,) dying of the typhoid affection. The symptoms occurring in infancy are closely related; the chapters on diagnosis, etiology, and treatment are considerably enlarged.

With the collection of eighteen cases we need detain the reader no further than to recommend them to his notice, as models of what such cases should be, as prototypes of those we should desire ourselves to bring forward (were this possible) either in substantiation or in refutation of the application of the general inferences from them, to the disease existing in these isles. The histories of 32 other fatal cases are interspersed through the work and from 46 of the 50 thus reported the general anatomical description is derived. Of these 46 cases:

| | | | | |
|---|---|---|---|---|
| 10 | terminated fatally from the | 8th to the | 15th day : | 1st period. |
| 7 | .. | 16th .. | 20th .. | 2d .. |
| 20 | .. | 20th .. | 30th .. | 3d .. |
| 9 | .. | after the | 30th .. | 4th .. |

The lesions of the digestive organs are first examined. The pharynx was the seat of morbid change in 8 cases ; of ulceration in 6, of submucous purulent infiltration, or of false membrane deposited on its free surface in 2. In no instance were the follicles of the pharynx (M. Chomel's experience is to the same effect) diseased in the manner of the crypts of the intestine. The ulcerations were not found in any subject dying before the 15th day, and in one case only proving fatal after the 30th. In 70 subjects dying of other acute diseases, exclusive of variola, no single instance of pharyngeal ulceration occurred; a fact which though not sufficing to prove such lesion peculiar, among all acute diseases (except smallpox), to the typhoid affection, yet demonstrating its great diagnostic importance in, and entitling it to the rank of a secondary lesion of, that disease.

Similar ulcerations existed in the œsophagus in seven cases, generally attended with some more or less marked lesion of the mucous membrane of the stomach, in no instance having led to perforation, and observed in such subjects only as died after the 16th day. No such ulceration was discovered in the victims of other acute affections; though extensive destruction of the mucous, with softening and attenuation of the submucous, tunic (a lesion terminating in perforation of the œsophagus in two cases of typhoid fever, more recently observed by M. Barth,) occurred in three instances. The inference drawn in respect of pharyngeal common ulceration, is clearly applicable to the œsophageal also.

It results from the general comparison made by M. Louis, in respect of the mucous membrane of the stomach, that this tissue was in the two orders of fatal cases (typhoid and common acute) affected with the same species of lesions, and in very nearly equal proportions. Thus softening and attenuation were observed in $\frac{1}{5}$ of the typhoid cases and in $\frac{1}{6}$ of those dying of equally acute complaints; ulceration in $\frac{1}{12}$ of the former, and $\frac{1}{24}$ of the latter; simple softening in somewhat less than $\frac{1}{6}$ of the first, and in $\frac{1}{7}$ of the second category of patients; mammillation inversely in about the same ratio; lastly, the mucous membrane presented its normal characters in $\frac{2}{7}$ ($\frac{10}{35}$) of the typhoid subjects, and in $\frac{1}{5}$ ($\frac{7}{35}$) of the others. Upon these important facts hinges the total subversion of the gastric theory of Broussais; they as clearly demonstrate that the typhoid fever of France is not a *gastro-enteritis* as induction can. They show that it would be just as rational to term pneumonia a *gastro*-pneumonia, because affections of the gastric lining membrane occasionally arise in its course, as to call typhoid fever a *gastro*-enteritis. But further, in consequence of the multiplicity of the diseases, during which such gastric complications are wont to be developed, and of the fact that such development does not take place until a more or less advanced period of the original malady, there follows one of the most legitimately deduced and most important laws of diseased action, thus stated by its discoverer: "In a certain number of cases in which an acute affection, of whatever nature this may be, gives rise to a febrile movement of any duration, the mucous membrane of the stomach becomes, at a variable period of that affection, the seat of an anatomical change proportional in degree to the predisposition of the subject; a change which hastens to a greater or less extent the fatal termination of the case, and may in some instances be the true cause of death."

It is unnecessary to notice particularly the intimate characters of any of the lesions of tissue referred to, with the exception of softening and attenuation attended with destruction. M. Louis, grounding his judgment on the absence of signs of inflammation around the softened part and on the coexistence of softening and attenuation of the submucous tunic—this too without surrounding evidences of inflammation—a phenomenon not observed in undoubted cases of inflammation of mucous tissues, questions its inflammatory character. He is inclined to admit that it may depend in some cases (as Dr. Carswell has shown to be the fact in rabbits) on dissolution by the gastric juice; but demurs to the general application of that distinguished observer's results to the human subject. His argument, drawn from the fact that death occurs occasionally in the human being under circumstances which would in animals infallibly entail dissolution and probably perforation, without any such effect being induced (an argument already very naturally applied by Mr. Taylor with similar intention),* incontrovertibly disproves the justness of such general application. It also shows that in cases wherein the human stomach appears to undergo the species of chemical change in question, there are some subsidiary conditions necessary to its production, not yet understood. M. Louis makes it manifest that these are probably unconnected with the state of the surrounding atmosphere, as such presumed dissolution occurs in the same proportion of cases in hot and cold weather. Mr. Taylor maintains that they are referrible to a morbid state of the gastric juice itself, a notion to which some obvious objections may be raised.

The mucous membrane of the duodenum was affected in fourteen out of twenty-two cases; discoloured in six, grayish in two of these in which death occurred at an advanced period of the affection; studded with one or two small superficial ulcerations in two cases. With the exception of the non-existence of the latter lesion, this portion of the intestinal tract

* Vide Brit. and For. Med. Rev., vol. IX. p. 375. April, 1840.

4289

presented the same species of changes in the typhoid as in the other category of subjects.

Enlargement of the caliber of the small intestine was detected in fourteen out of thirty-nine typhoid subjects, exclusive of those dying from perforation of the ileum; and appears to be more frequent in the earlier periods than in the fourth,—a fact, showing that meteorism (the cause of the enlargement in question) cannot be directly ascribed to ulceration of the bowel. Invagination of the bowel occurred in three cases, without giving rise to any particular symptoms. Ascarides were found in a certain but undetermined number of cases; there was always a greater or less quantity of mucus in the intestine except in one case of perforation; and commonly an abundant supply of fluid bile. An orange tint in the neighbourhood of red spots appeared traceable to exhalation of and subsequent changes undergone by blood.

The colour of the mucous tunic varied with the period at which the disease had proved fatal. It was white in

| | | | | |
|---|---|---|---|---|
| 4 | out of | 10 | subjects of the | 1st series |
| 3 | .. | 7 | .. | 2d |
| 5 | .. | 20 | .. | 3d |
| 1 | .. | 9 | .. | 4th |

in other words the earlier the occurrence of death, the stronger the chance, as might have been anticipated from the facts already disclosed, of finding its hue natural. The converse is true of *gray* discoloration: this was observed in only two subjects among those who succumbed before the twentieth day, in seven of those who perished from the twentieth and thirtieth, and in four of those lingering beyond that period. In a third part of the cases the surface was red; a yellow tinge, evidently from bilious stain, appeared in four instances only. It follows then that the gray hue is almost peculiar to the intestine of subjects dying after the twentieth day; and that this is a transformation of the red, the observed condition of the parts essentially implicated in the disease appears, as we shall presently see, to demonstrate.

The mucous membrane (excluding from this statement that of the patches of Peyer) was of natural consistence in nine out of forty-two subjects, and in a greater proportion of individuals dying after than before the 30th day. It had lost its natural share of consistence to a greater or less degree in all the other cases, and in the following proportions in the different periods:

| | | | |
|---|---|---|---|
| in 7 of the | 1st series | in 16 of the | 3d series. |
| 5 | 2d | 5 | 4th |

The secondary character of this softening clearly appears from its absence in a number of the subjects whose decease occurred from the 8th to the 15th day of the affection; but its intimate nature is far from being equally manifest. A luminous enquiry into the point, based on observed facts, leads the author to the following conclusion, which we are induced to extract on account of its important bearing on a general question of pathological anatomy:

"Every consideration, consequently, appears in favour of the notion that simple softening of the mucous coat of the small intestine, unaccompanied with thickening or redness, is, at least in part, the result of incipient cadaveric decomposition. I say in part, because the existence of this softening in a certain number of subjects dying in the cold season of the year, shows that some other cause must also be in action,—whether this be some, as yet unascertained, lesion of the mucous tunic itself, or an altered condition of the liquid and other contents of the bowel."

The agminated glands, patches or glands of Peyer, were more or less diseased in every case, through an extent of the intestine varying from two to eight feet. This lesion presented itself under two distinct aspects, distinguished by the epithets of *soft* and *hard.* To commence with the former: The most advanced changes invariably appeared in the patches nearest the cæcum; a sound patch rarely appeared between two diseased ones, and the transition from the morbid to the natural state was commonly sudden. The patches affected with the least marked disease were but slightly prominent, and of delicate pink hue; their mucous membrane slightly softened; the orifices of the crypts imperceptible. These different characters assumed more marked development in the adjoining patches, the free surface of these appearing finely mammillated, the orifices of the crypts manifest, the corresponding submucous tissue more or less thickened and red. In a still more advanced stage the redness, thickening, and softening of the tissues implicated, had acquired further development; mammillation ceased to be visible, nor could the mucous tunic, as in the earlier stages, be removed from the subjacent tissue, which had itself meanwhile grown thicker and redder than before. Nearer the cæcum ulceration exhibited itself, at first superficial, occurring in a single or in several points, sometimes of very small size, in other instances producing total destruction of the mucous coat of a patch, either through the union of several minute ulcers, or extension of a single one. The ulcerative process successively destroyed the submucous, muscular, and peritoneal tissues, leading to complete perforation in eight out of fifty fatal cases. The form of these ulcerations is not characteristic; it may be round or oval, its edges smooth or denticulated, vertical or bevilled off. These lesions were most prominently marked in subjects dying between the 15th and 30th days; any ulcerations discoverable before that period were generally small, and few in number, or, as was the case in two instances, these were absent altogether,—redness, softening, and thickening constituting the only morbid changes therein. This absence of ulceration was, however, also established in two cases terminating fatally, at so advanced a period as the 25th and 29th days; a very striking exception to the ordinary course of things. The usual appearances discovered after the 30th day indicated a retrograde action in the patches: the strong red hue had given place to one less marked and mixed with gray or blue, while the tissue itself had become firmer, and lost much of its abnormal thickness; at a still later period the edges of the ulcerations were sunken, and their field covered wholly or partially with a shining, polished, and extremely delicate pellicle of serous aspect, and continuous with the submucous tissue beyond the confines of the ulcer. These evidences of cicatrization were never detected before the 37th day of the disease, and always in the immediate vicinity of the cæcum; reparation commenced where the diseased action had first set in.

The *hard* patches were distinguished from those we have described by the condition, not of the mucous but of the submucous tissue. In this variety of the disease the latter tissue was "transformed" into a homogeneous matter, without apparent organization, of a pale pink or yellowish tint, presenting a dry and shining surface on division. Before ulceration the surface of this variety of patch is plane and uniform; after that change the patches become more or less deeply furrowed and uneven. The portion of abnormal stratum in contact with the mucous membrane is soft and friable; its deeper layers possess the consistence of the lymphatic glands. Thirteen of the 46 subjects examined furnished examples of this variety of patch; in three cases it coexisted with the other. The hard species occurred with much greater proportional frequency among subjects dying from the 8th to the 15th day, than at a later period; and in no instance was it discovered, where death had supervened after the 30th day. The inference M. Louis felt inclined to draw from these facts as to the more fatal character of the form of lesion now under consideration, is supported by the subsequent observation of M. Barth, at the Hôtel-Dieu: this physician found the mean duration of the disease to be only 19 days in 8 cases of the hard, 36 in 19 of the soft, variety. Reasoning upon the invariable coexistence of the lesion of the submucous with that of the mucous tissue under all circumstances, M. Louis concludes that both become affected simultaneously; a consideration sufficient in itself to demonstrate the specific character of the disease, as in ordinary cases of mucous inflammation the subjacent tissue does not, in the majority of instances, participate in the inflammation. The fact that the morbid change of the cellular tissue of the hard patches had in several instances attained a very marked degree of advancement, while that of the investing mucous tunic was only moderately softened, seems to warrant our entertaining the belief, "that in a certain share of cases the alteration of the cellular commences before that of the corresponding mucous membrane." M. Chomel goes further than this, considering himself enabled, by his own observations, to affirm that, in some instances at least, the disease distinctly originates in the tissue beneath the mucous coat. And though the legitimacy of his conclusion is fairly questioned by M. Louis, inasmuch as his premises do not appear to warrant it, still that it is a correct one as applied to other facts is rendered probable by the microscopical investigations of Boehm, on the agminated glands in the "abdominal typhus" of Germany. "The first and evident change," to use this observer's language, "(as Carus has found,) consists in an exudation, which takes place in the submucous tissue . . . Thus the inflammation of the intestine [more correctly of the *mucous membrane of the patches*] is not to be considered as *primary*, but as *consequent* on the distension and irritation of the mucous membrane of the corpuscules covering the exudation . . . The mucous membrane being destroyed, the bottom of the ulcer is formed by the exuded matter, which is dense, lardaceous, hard, often rough, uneven, furrowed, and surrounded by callous margins often of a peculiar yellow colour."* Notwithstanding the counterargument urged by M. Louis to the effect that in one case proving fatal from perforation on the 36th day, the submucous tissue, "though thickened, retained its ordinary white colour," we think it may be accepted, not certainly as a demonstrated fact, but as a very likely

* Vide Brit. and For. Med. Rev., vol. I. p. 524. April, 1836.

one, that the hard form of the disease originates in the submucous tissue.* M. Louis differs from M. Chomel in regarding the lesion of both varieties of patch as identical, in regard of the mucous membrane; nor does he consider the lesion of the latter tunic in the soft patches as essentially gangrenous, though gangrene may supervene as a termination of preceding inflammation. Reasoning upon the presumed gangrenous nature of this lesion, M. Chomel concludes that the hard patches only are susceptible of resolution, but our author correctly observes that the cases, adduced in the work of that observer, as proving the reality of such susceptibility on the part of these patches, are very far from establishing the point.

The solitary glands were more or less diseased in twelve cases, and with one exception, the changes affecting them were most advanced in the vicinity of the cæcum.

From a review of the lesions observed in subjects dying of other acute diseases, it follows that, setting aside the abnormal state of Peyer's glands of which not a single example occurred except as an attendant upon the typhoid affection, the morbid changes in the small intestine were the same in nature, and very closely so in proportional frequency of occurrence, as in those who succumbed to the latter malady. Hence the inference that the law already explained as regulating the development of gastric lesion during protracted febrile action prevails also in regard of the small intestine.

The caliber of the large intestine was enlarged in consequence of meteorism in twenty-two out of thirty-nine cases; in three fourths of these the distension was so considerable as to obstruct the actions of the thoracic and abdominal viscera, and occasionally drive the liver considerably beyond its natural level superiorly. This distension of the intestinal walls, not traceable to any lesion of the mucous membrane, considerably more frequent in subjects dying between the 20th and 30th days, than before or after that period, was attended with thickening of the muscular coat; a fact illustrating the rapidity with which muscular hypertrophy may be established, and sufficing to prove, independently of any other phenomena, that the development of gas had occurred during life. The mucous coat was in four instances studded with *hard* patches of the same character as those of the small intestine, but of much smaller size, their diameter varying from three to four lines, non-friable on the surface, and in one instance only ulcerated superficially. Ulcerations, to the number of two or three in each instance, varying in diameter from four to thirty lines, capable of cicatrization, as ascertained by M. Barth in two cases of death on the 43d and 120th days, presented themselves in fourteen subjects, in the following proportions in each series:

| | | | | | |
|---|---|---|---|---|---|
| 1 | case out of | 10 | of the | 1st | series. |
| 2 | .. | 7 | .. | 2d | .. |
| 9 | .. | 20 | .. | 3d | .. |
| 2 | .. | 9 | .. | 4th | .. |

With the exception of these hard patches the lesions discoverable in the large intestines of subjects dying from all other acute affections were of the same description as in the instance of typhoid patients; nor did the proportion of these, except as regards meteorism and ulceration, mate-

* This must be understood to refer to subjects of fifteen years of age and upwards alone.

rially differ in the two categories: this appears distinctly from the subjoined table:

| | | Deaths. 45 from typhoid fever | Deaths. 69 from other acute diseases. |
|---|---|---|---|
| Mucous Membrane. | Universally red | 3 | 3 |
| | Partially red | 10 | 12 |
| | Grayish | 9 | 7 |
| | Universally softened | 16 | 22 |
| | Partially softened | 14 | 25 |
| | Ulcerated | 14 | 3* |
| The bowel tympanitic | | 25† | 2 |

The lesions of the abdominal lymphatic system are in this affection marked and characteristic. The mesenteric glands corresponding to the diseased patches were invariably diseased; in the early stages enlarged, red, and softened, at a more advanced period infiltrated or studded with specks of pus. In some individuals whose death had taken place between the 20th and 30th days, the red discoloration had given way to a grayish hue, and the glands lost, to a certain extent, their abnormal enlargement and softening,—direct indications of their being on the return to the normal state. The mesenteric glands corresponding to healthy patches presented changes of the same kind, but less marked, in $\frac{1}{4}$ of the subjects. The mesocolic were the seat, but not in every instance, of an analogous form of lesion; the cervical and gastric occasionally red and enlarged; those of the biliary ducts intensely inflamed in 2 cases. In other acute affections the condition of these parts was extremely different, and from the facts now adduced the following propositions very clearly follow: 1. The lymphatic glands may become diseased through the influence of causes existing in the system generally, independently of the mucous membranes, with which they are in physiological connexion. 2. The latter are frequently diseased without implication of the corresponding glands, although the lesion of the former may have existed for some time before death. 3. Softening and enlargement of the mesenteric glands, when existing to any extent, constitute a lesion proper, among acute diseases, to the typhoid affection, and are of almost as great importance in this point of view as the lesion of Peyer's glands themselves. 4. There is a special tendency to disease of the lymphatic glands generally in the typhoid affection. The importance of the first two propositions springs from the refutation they convey of some favorite dogmata of Broussais.

Of hardly less constant occurrence than the affections of the patches of Peyer and of the mesenteric glands is a peculiar morbid condition of the spleen: in four cases only was this organ healthy, twice in subjects dying between the 20th and 30th days, twice in cases terminating fatally after that period. The organ was ordinarily enlarged and softened, in many instances had attained four or five times its normal size; and was invariably much enlarged when the additional lesion, softening, existed to a considerable amount. Different varieties of discoloration coexisted with these changes. Now, inasmuch as the four exceptional cases occurred among individuals dying after the 20th day, and the organ was

* This is of course exclusive of dysentery.

† The observed number 22 out of 39 is here raised to the ratio of 45.

never found healthy before that period, it becomes exceedingly probable that the spleen is in reality implicated in every case at an early period of the disease, a notion confirmed by the semeiology of the malady. Respecting the nature of these combined lesions,—while he rejects the notion of their being inflammatory on account of, 1st, the invariable absence of pus ; 2dly, the fact of the splenic parenchyma being *universally* and *equably* affected throughout, (a singularly uncommon state, if indeed it ever arise, of parenchymata confessedly inflamed ;) and 3dly, the invariably sound condition of the investing membrane of the organ,—M. Louis shows it to be impossible, in the existing state of knowledge, to draw any determinate inference.

In thirty-two only of eighty-four individuals dying of other acute diseases was the spleen notably affected, and in these cases the changes it had undergone were much less marked than in typhoid subjects. Particular ages appear to be without influence on the development of this lesion, nor is any connexion traceable between it, the existence of diarrhœa or of disease of the stomach or of the large intestines.

Continuing the examination of the abdominal viscera we come to the liver, an organ softened in 22 of 46 typhoid cases, commonly throughout its entire extent, and at the same time pale and friable,—changes of equally frequent occurrence at all periods of the complaint. And the inferior importance of this lesion, as exhibited in the comparative rarity of its existence, is thrown into stronger relief by the consideration of the circumstances under which it was observed ; from these it appears that, though in some measure dependent on some predisposing condition of the solids or liquids, the softening was materially influenced by physical agency, especially by temperature. The changes discovered in the liver of the other category of subjects, resembled in frequency of occurrence and in character those of the typhoid class; a somewhat greater frequency of softening in the latter being the only notable distinction. Curiously enough, emphysema of the hepatic tissue was detected in three instances, coinciding with similar development of gas in the common cellular membrane, while not a single example of the former phenomenon presented itself among the typhoid subjects.* The liver was fatty in two patients, one dying of peritonitis, the other of an indeterminate affection.

The bile was very abundant, fluid, reddish or greenish, in the majority of typhoid cases, and sometimes turbid ; the gall-bladder contained pus in three instances, in which its mucous membrane was red and thickened. The same law prevailed here as in regard of the organs generally, in respect of the period at which the most advanced disease exhibited itself; they were more serious when death occurred at an early than at an advanced period. In the victims of other acute affections the lesions of the biliary apparatus were of the same character, but of much less frequent occurrence than in the typhoid class.

Enlargement, softening, and violet-red discoloration of the kidneys existed in a few subjects, most marked in those cut off at an early period. The same modes of lesion were discovered in other subjects, not however with the same frequency or to the same extent. In commenting upon

* That such emphysema sometimes occurs in the typhoid fever of Paris, at least as a post-mortem phenomenon, could scarcely be doubted à priori, and is shown to be the fact by M. Chomel's cases; but this does not diminish the interest of M. Louis' results.

the statements of M. Rayer regarding the development of nephritis in typhoid fever, M. Louis observes that the general law of the greater frequency of secondary inflammatory lesions in that malady suffices to explain the fact, though he does not deny the influence of retention of urine upon its development. This is hardly different from the doctrine laid down by M. Rayer himself, as may be seen by referring to his second volume, or to the review of it in this Journal. (Vol. X. p. 302.)

Two typhoid subjects were manifestly affected with pyelitis; the mucous membrane of the pelvis of the kidney being red, thickened, and bathed in one of them with pus. The only remarkable point in the condition of the bladder, besides its being extremely distended with urine in a fifth of the subjects, was the existence of a minute ulceration in one case. One typhoid subject only had suppurative inflammation of the parotid glands, the affection of these parts was equally rare in other acute diseases; the pancreas did not in any instance present notable deviation from the normal state.

We shall pass more rapidly over the lesions detected in other organs, these having on analysis been demonstrated to be without intimate relation to the phenomena of the disease. The heart was healthy in somewhat more than half the cases, more or less softened in the others, and sometimes to an extreme degree. Under these circumstances its tissue was of livid colour, its walls attenuated and easily torn, its contents a few drops of blood, mingled with air or clotted and non-fibrinous. M. Louis' conclusion as to the cause and mode of production of this softening, agrees precisely with that already explained in respect of the similar change observed in the liver. In almost every instance wherein the heart appeared softened, the internal surface of the aorta was tinged red, and its lining membrane occasionally softened and thickened; a phenomenon produced by imbibition, favoured by some peculiar non-inflammatory condition of the blood or arterial tissue, and in some instances probably setting in prior to death. The discoloration in question rarely occurred, and was always trifling in amount, when the heart retained its normal characters.

Redness, peripheral thickening, and investment with false membrane existed in the epiglottis in two cases; partial destruction, combined with thickening, in the sixth part of the subjects; the larynx was lined with false membrane in three instances, the seat of a small ulceration in one other. M. Louis found the lungs healthy or nearly so, in somewhat less than half the patients, whose death occurred from the 8th to the 20th day, and in somewhat more than the fourth part of those who died at a more advanced period; in a state of splenization or hepatization, or of both combined, in all the others. The pleura contained sanguinolent serosity in nearly half the cases. All these abnormal states were observed with very nearly the same frequency in subjects dying of other acute disorders, with the exception of course of pneumonia.*

The arachnoid contained two or three small spoonfuls of transparent serosity in three cases, of turbid in one; in another instance a soft, ex-

* Not of pleurisy, as the reader might perhaps expect us to add. Our author has shown that pleurisy rarely, if ever, proves fatal, unless in subjects labouring under chronic disease; such subjects are of course excluded from consideration throughout the present comparisons.

tremely recent pseudo-membrane. The subarachnoid cellular tissue was more or less infiltrated in 28 subjects,—to any considerable extent in 4 only, and in 3 the phenomenon was distinctly ascribable to the unusual duration of the "agony." The pia mater was injected in somewhat less than half the cases, to a remarkable degree in 11 subjects; more frequently in those dying from the 8th to the 20th day, than after that period; the superior cerebral veins distended with a tolerably large quantity of blood in one fifth of the cases. The cortical substance of the cerebrum was more or less red or pink coloured in 17 cases; the medullary injected to a moderate extent in the majority of subjects. Both were slightly softened in 7 instances; there was local softening in two others. The cerebellum presented the same species of lesions in a less number of cases. The injection of the pia mater and medullary substance, the pink hue of the cortical substance, and undue firmness of the entire mass, are more frequent and more marked, the more rapid the death of the subject: the contrary was the truth with sub-arachnoid infiltration, effusion of serosity into the lateral ventricles and softening. Of the non-inflammatory nature of these lesions no question can be raised after the study of the acute enquiry into the point by M. Louis; the length of this prevents us from extracting it entire,—to condense would be to mutilate.

Whatever importance the reader may feel inclined to attach to these conditions of the encephalon as connected with the symptomatology of the disease, the fact that similar cerebral conditions were observed with almost the same frequency in persons dying of all other acute affections indiscriminately, exhibits clearly their insignificance in this point of view. The only condition of the viscus apparently peculiar to typhoid fever—namely, a certain viscidity of tissue existing in one half the cases—has no claim, in the present state of knowledge at least, to consideration.

The remains of erysipelas existed in 4 subjects; the skin was thickened or attenuated, partially ulcerated or completely destroyed where blisters had been applied. In one instance only was the cellular membrane emphysematous, whereas it was so in 8 of the non-typhoid class of individuals. This latter phenomenon is by M. Louis ascribed in considerable measure to a morbid state of the fluids, favorable to the establishment of putrefaction; if this be the case, it would follow that the state in question is less frequent in typhoid fever than in other acute diseases.

Such then are the anatomical changes observed in subjects dying with the group of symptoms constituting the typhoid fever of Paris,—such their frequency, such their degree. Reviewing these we find that the sole lesions of invariable occurrence are seated in the patches of Peyer and mesenteric glands; is there any other legitimate conclusion from this fact than that these lesions are, in the language of the author, "inseparable from the existence of the affection under consideration, and constitute its anatomical character?"* And further, as the lesion of the

* We need scarcely point out to the reader that this conclusion differs *toto cœlo* from that almost invariably ascribed to M. Louis in this country,—namely, that the affection of the patches of Peyer is the *cause* of typhoid fever. The one inference is not only just, but a manifest involution of the premises; the other utterly unwarranted thereby, and unworthy of the humblest follower of inductive medicine. There are some minds of such curious perceptive power that they are unable to comprehend the wide difference between *cause* and *anatomical character*, and who, we presume, would charge any pathologist maintaining that the pustules of smallpox constitute the anatomical character of that disease,—in other words, the only material evidence of its existence,—of ranking them as its cause. With individuals of such mental caliber it is vain to argue.

agminated glands had acquired a certain degree of intensity in subjects opened on the 8th day of the disease; as in the greater number of cases the initiatory symptoms indicated a morbid change in the intestinal canal, (while the fact of the comparatively slight implication of the *general* mucous tract excludes the idea of their having originated in the changes developed in it,) the inference is, that the affection of Peyer's glands commenced with the first functional derangements. And the characteristic value of this particular lesion is thrown more prominently forward by the fact that the great majority of others occurred, as we have seen, with similar frequency in the course of acute disease generally.* The only exception of consequence to this statement appears to arise from the very frequent development of ulceration in the membranous tissue of typhoid subjects—a phenomenon occurring with singular rarity in other acute maladies, and hence forming a distinguishing feature of the disease, while it justifies the view of M. Louis, that "typhoid fever bears in this respect the same relation to other acute diseases, that phthisis does to chronic."

The cases upon which this anatomical doctrine is based, having been collected at an hospital from which individuals under 16 years of age are commonly excluded, it could not justly be applied to the disease as existing in children. Direct observation had however subsequently proved beyond doubt that the same mode and form of lesion of the intestinal glands prevail before as after the sixteenth year; and the recent extensive and minute investigations of MM. Rilliet and Taupin, at the Hôpital des Enfans, have demonstrated not only the general identity of the morbid condition of the glands, but the close similarity of the secondary lesions developed in the infant malady. These secondary lesions, which we have in the greater number of instances distinctly pointed out, depending upon the continuance of febrile action, the obvious inference is, that the existence of an inflammation entails that of other lesions, inflammatory or otherwise. Now the importance of this law in respect of the natural history of disease will, we believe, be questioned by none; but has it no practical application? has it no reference to the practice of revulsion and cauterization in acute diseases,—modes of treatment to which certain persons are wont to ascribe most marvellous effects? To us it appears to show that the alleged efficiency of agents of the class in question may fairly be disputed. And in this it appears to corroborate the results of direct clinical experience; for our own parts we are disposed to affirm that there is not a single one of the class of *acute* visceral inflammatory affections in which blistering and other modes of counter-irritation have been *demonstrated* to exercise a salutary therapeutical influence,—that is, to lessen the mortality produced by, or diminish the mean duration of, any malady referrible to that category.†

* Although in the extensive series of cases which fell under the observation of M. Louis, no example presented itself of the special lesion of the agminated glands, except in cases of typhoid fever, yet we do not mean here to prejudge the question of the possibility of such occurrence,—this will come to be discussed in a future article.

† We have elsewhere (Brit. and For. Med. Rev., vol. IX. p. 317,) noticed one of the probable causes of the exaggerated idea of the utility of blisters in certain affections. We should be glad to find that we have in our turn fallen into the opposite error of undervaluing them; but we may premise that our conviction of being correct—founded as it is upon the accurate investigations of unbiassed observers,—will not be shaken by the outcry of persons arrogating to themselves the title of "practical," who, if they have not cured all manner of maladies with blisters—we admit this beforehand—have no doubt employed them, in current phrase, with the *"happiest effects."*

There is yet another aspect in which these secondary lesions assume pathological importance. If it be true that in the great majority of cases individuals who die with a group of symptoms confessedly referrible (not as to a cause but as to an observed coexistence,) to a given physical change of tissue—their anatomical character—the extent, nature, or seat of this morbid change, suffices to explain the decease of the individual; it is no less certain that in many instances this is inadequate to account for the observed issue of the disease. In these instances death may be either fairly ascribed to secondary lesions, or the event, as occasionally happens, is not to be explained by the gross physical changes discoverable in all the tissues collectively. Now in 18 of the fatal cases of typhoid fever (two-fifths of the whole) the condition of the patches of Peyer, of the intervening mucous membrane and mesenteric glands, was insufficient to account for death; and in 14 of them the event was traceable to the secondary lesions; while in the remaining 4 even these failed to explain it. Facts of the latter exceptional order have been appealed to with no small exultation by those who have satisfied themselves with a superficial knowledge of M. Louis' doctrine, as subversive of the importance attached by him to the Peyerian lesion. No better example could be adduced of the necessity of collecting a large mass of facts of all descriptions, before the attempt be made to determine the value or bearing of the phenomena of any individual case. The assumed importance of the disparity of the lesion and symptoms sinks into insignificance in respect of the notion which it is appealed to to support, on the announcement that a similar disparity was detected in the series of cases compared with the typhoid,—that in 5 out of 34 cases of pneumonia (to take a single example) the pulmonary lesion was insufficient to account for death, and in two of these the secondary lesions similarly ineffectual. And if the fact be taken into consideration that in several instances the typhoid lesions had commenced, or even made considerable progress in, a retrograde course, the proportion of cases in which the fatal issue appears a natural result of the state of the organs, will equal at least that observed in other acute diseases.

We have now reached the third part of the work, devoted to the symptomatology of the malady. Here M. Louis, having commenced with a general view of the symptoms, proceeds to make each of these the subject of separate examination. The time of origin, duration, and intensity of each; their relation to the different lesions discovered in *fatal* cases, are successively established; and then the same series of enquiries instituted in respect of the symptoms of individuals, who having had the disease *severely* or *mildly*, ended by recovering. The same course is next pursued in regard of the symptoms attending other acute diseases. Our means will not permit us to do more than make the reader acquainted with the chief characters of the symptoms observed in typhoid fever: we shall consider together the symptoms of fatal, severe, and mild cases, as to follow the distinction of these three classes, established by M. Louis, would inevitably carry us beyond reasonable limits.

*Diarrhœa* occurred in 129 out of 134 cases; in 58 of 125 on the first day of illness; was very considerable in 36 of 120, and of a mean duration of 20·5 days in cases of recovery.

*Abdominal pain* was detected in 112 of 127 subjects, arose on the first day in 37 of 109, was commonly obtuse, of a duration varying from 24 hours to a month, and ordinarily seated in the iliac fossæ, especially the right, but in some instances affecting the abdomen generally.

*Meteorism* existed in 89 of 134 subjects, at some period of the disease was occasionally considerable, never appeared earlier than the seventh day, and lasted on an average from 4 to 15 days.

*Epigastric pain* was present in 59 among 96 patients; in 19 of 59 from the outset of the disease; in some subjects it was doubtful whether this was seated in the stomach or colon.

*Nausea* occurred in 37 of 95 patients, on the first day in 11 of 37; varying in duration from a few moments to 20 days.

*Vomiting* occurred in 29 among 94 subjects; in 7 of 28 on the first day.

*Cephalalgia* was noted in 127 out of 134 cases; appearing from the first in 39 out of 42 fatal cases, and in 74 of 85 subjects who recovered; the mean and ordinary duration being 9 days; it was violent in a very few instances only; in the rest it scarcely engaged the attention of the patients.

*Somnolence* exhibited itself in 109 of 134 cases, rarely before the sixth day; was slight at the outset in all; in 10 of 40 severe cases of recovery strongly marked; in these cases its mean duration was eight days; with hardly an exception it appeared before delirium.

*Delirium* was noted in 70 of 133 cases, and rarely commenced before the eighth day; ran a mean duration of ten days in fatal cases, of 6·5 days in severe cases terminating by recovery.

*Epistaxis* appeared in 49 out of 74 subjects; in 6 of these on the first day.

*Tinnitus aurium* was experienced by 36 out of 99 subjects, in 7 only on the first day.

*Deafness*, occurring commonly towards the middle or close of the disease, was manifest in 38 among 99 patients; in the worse order of cases was sometimes extreme, and varied in point of duration from one or two to twenty days.

Of 114 subjects in whom this was looked for, 91 presented a *peculiar eruption*, consisting of small florid papular elevations of lenticular shape, disappearing under pressure, few in number in the majority of cases, principally observable on the abdomen, but when abundant exhibiting themselves on the limbs; developed first, in all probability, between the sixth and ninth days; one set of papulæ disappearing and giving place to another, the whole running a mean course of about eight days.

*Sudamina* made their appearance in 28 of 43 cases, never being observed before the twelfth day; their diameter varied from two to four millimeters; they were produced by elevation of the epidermis with a colourless transparent fluid, and their development observed no distinct relation to the intensity of the sweats.

*Erysipelas* declared itself in 6 of 46 fatal cases, in 3 of 57 severe cases of recovery, in none of those of mild character.

The *Pulse* varied in character; thus of 41 subjects who died, 13 had a large and full pulse till the closing days, 8 had it remarkably large, 20 small; in respect of quickness it averaged 80-96 in 8, upwards of 100 in the rest; in 7 it was intermittent, in 34 regular.

*Buffy blood* was observed in 5 of 12 subjects who died, and were bled; in 1 of these the buff was firm, thick, and semitransparent; in the others soft, gray, and gelatiniform. In 8 of 32 cases of recovery the blood was buffy; in 1 the buff thick, yellow, and transparent; in 1 dense and red, in 6 yellowish, and commonly thin, soft and gelatiniform.

It appears that the duration and intensity of the diarrhœa corresponded with those of the disease. The matters discharged were commonly thin, rarely containing small pieces of solid substance, a little mucus in four cases only; in two subjects they resembled coffee grounds; two other patients discharged some blood temporarily. The latter description of stool being of singular rarity in other acute diseases, with the exception of dysentery, its occurrence *may* become useful in a diagnostic point of view, though it is probable that when it arises, the other symptoms will be sufficiently characteristic. In respect of other acute diseases, this intestinal symptom confirms the result already referred to respecting the implication of the digestive tube in all febrile affections.

The objection which might be raised to the notion of early development of the intestinal lesion on the score of the absence of pain at the outset in a certain number of cases, M. Louis considers to be refuted by the fact that pain is not a constant attendant upon inflammation, in cases where this is even *seen* to exist. Thus, he has observed inflammation of the mucous membrane of the velum palati and of the tongue, erysipelas, &c. unattended with pain. He maintains accordingly that pain "is one of the least constant symptoms of disease, whatever be its seat, and one of the least calculated to throw light upon the diagnosis; nevertheless the period of origin of disease may in a considerable number of instances be inferred by its aid with precision, and in this respect it assumes much importance."

Upon the cause of meteorism the facts under review appear to throw no light; that it cannot be attributed to putridity follows from its having decreased in several subjects during the last days of existence; nor can it, at least exclusively, arise from alteration of the properties of the blood, as such alteration attends numerous acute diseases unaccompanied with meteorism; nor, we may add, can it depend upon decomposition of the feces, for the small intestine is its chief seat, whereas these sojourn in the large. The phenomenon appears to possess somewhat of a specific character.

The study of the gastric symptoms in the different classes of affections observed by M. Louis shows that, in the first place, affections of the mucous coat of the stomach often run a latent course during acute disease, a fact confirming the importance attached to the law respecting the development of lesions of the alimentary canal under these circumstances. Acquaintance with this law teaches us to be constantly prepared for the development of complications materially modifying the prognosis, while symptoms give us no hint of their advent. From M. Louis' cases we also learn that epigastric pain, coupled with bilious vomiting, almost invariably announces a serious lesion of the mucous membrane of the stomach; and that such lesion generally arises at an advanced period of the affection. On the other hand neither epigastric pain, nor even such pain attended with nausea, are infallibly indicative of an appreciable change in the tissues of the stomach, although they are in some instances the only observed effects of such change. This disclosure of the diagnostic inefficiency of epigastric tenderness, made at a time when the speculations of Broussais were cherished by the crowd, may be readily conceived to have excited notable indignation. Nor was this lessened by the added statement that the very affection upon which Broussais based his system—gastritis—is one of those with which pathologists are most imperfectly acquainted,—for this very natural reason that simple gastritis, terminating by the death of the individual it attacks, is of exceedingly rare occurrence, to such a degree that during a period of sixteen years, in which he has examined more than 1300 bodies, M. Louis has not met with a single example of that form of the affection. If it be true that the lesions of gastritis may be more frequently studied on the dead subject as secondary developments, it is equally certain that the examination of these had, before the investigations of M. Louis, been rarely, carelessly, and in fact but accidentally, attempted.

The conditions of the tongue and of the gastric surface in the fatal cases are exhibited in the subjoined table, which we have formed from the author's facts:

*Thirty-six Fatal Cases.*

| MUCOUS MEMBRANE OF THE STOMACH. | TONGUE | | |
|---|---|---|---|
| | Natural in 16 | Generally dry, bright red at the edges, reddish in the centre, rarely brownish in 12 | Dry, with crusted fur, brownish, more or less hard, in rare instances bright red in 8 |
| Softened and attenuated | . . . . 2 | . . . . . . . . 2 | |
| Mammillated with or without ulceration, ordinarily softened, thickened, or discoloured . | . . . . 9 | . . . . . . . . 5 | Thickened and mammillated without softening . . . . . 1 |
| Ulcerated . . . . | . . . . 1 | . . . . . . . . . | . . . . . . . . . 1 |
| Softened simply . . | . . . . 2 | With red or brownish punctuation . . 3 | With redness . . . 4 |
| Healthy . . . | . . . . 2 | . . . . . . . . 2 | . . . . . . . . 2 |

In two instances the tongue was more or less thickened and furrowed, in two others covered with a whitish pultaceous exudation. From the above table some inferences of consequence are derivable: 1. The tongue retains its natural characters in somewhat more than a third of fatal cases of typhoid fever. 2. The tongue may be natural and the gastric surface profoundly diseased. 3. The tongue may present the most marked evidences of implication in the disease, and yet the stomach be found not only not seriously diseased, but on the contrary perfectly healthy. 4. The same description of gastric lesion coexists with all varieties of condition of the tongue; and let it not be supposed that the cases, wherein the tongue maintained its healthy attributes, were mild in respect of other symptoms; independently of their being fatal cases, the very contrary was the fact. The analysis by MM. Rilliet and Taupin of fatal cases in children, of cases of recovery, and of all other acute affections in the adult, confirms the induction here drawn of the reciprocal independence of gastric and lingual lesion,—certainly not one of the least important corrections of professed experience which researches on the numerical system have established.

The function of deglutition was seriously obstructed in 10 of the 46 fatal cases as follows:

| | | | |
|---|---|---|---|
| 2 of 10 | of the first series | 4 of 20 | of the third series |
| 2 7 | second | 2 9 | fourth |

In eight of these cases the occurrence of the symptom was easily explained by the state of the parts, these being the seat either of abscess, infiltration with pus, plastic inflammation, muscular hypertrophy, or ulceration. In the two other cases the inside of the mouth was not carefully examined during life, so that it cannot be considered certain, though this be probable, that the inability to swallow was in them purely nervous. It must not be forgotten on the other hand that advanced disorganization of the pharynx or œsophagus may exist without dysphagia: such was the fact in four cases; here however the presence of delirium accounted for the absence of the ordinary effect. It is worthy of note, as observed by M. Louis, that when delirious patients obstinately refuse drink, without being able to assign their motives, a lesion of the pharynx is almost invariably detected after death.

Cephalalgia, as appears from the facts referred to, is one of the most common symptoms of acute febrile disease of every kind; nevertheless as it is a more frequent, indeed an almost constant attendant on typhoid fever, and in the vast majority of cases, whether fatal, severe, or mild (113 out of 127), originates from the very outset of the disease, its absence at the outset of a febrile disorder, more especially if other symptoms of typhoid fever be wanting, may be regarded as a very probable sign that the affection is not typhoid.

The characters of typhoid somnolence are sufficiently distinctive. In two variolous patients this symptom appeared on the third day of the feverish state, and it is obvious that as the general symptoms are at that period the same as in many cases of typhoid fever, the malady might be easily mistaken for the latter. However as typhoid somnolence rarely (6 times in 134 cases) comes on before the fourth day, that of the variolous eruption, its occurrence previous to that day warrants a strong suspicion that the affection is of the eruptive class.

M. Louis enquires at great length into the question as to the reality of the alleged existence of an appreciable relation between the occurrence of delirium, and the condition of the brain or other organs. The conclusion in respect of the brain is in the negative, and for the reasons deducible from the following table:

| State of the brain. | 12 subjects Non-delirious, or so only for 24 hours, or during the last 2 or 3 days of life. | 12 subjects With the most violent form of delirium. |
|---|---|---|
| Cortical substance more or less pink . . . | . . . . . . 4 | . . . . . . 5 |
| Perfectly healthy . . . | . . . . . . 6 | . . . . . . 5 |
| Brain injected . . . | One thalamus slightly softened . . . . 1 | Very much so . . 1 |
| Brain less firm than in health . . . . | . . : . . 1 | . . . . . 1 |

And further, in two of these twenty-four individuals the brain appeared abnormally firm; one of the two had violent delirium, the intelligence of the other remained unaffected. Now as the anatomical conditions of the brain in the two categories of subjects may almost be said to have been identical, while the states of the intellectual faculties were diametrically opposed, the negation of any necessary nexus between lesion of the brain and delirium in typhoid fever is justly predicable. Close study of the symptoms in cases of recovery corroborates this inference. Among other points of evidence in this direction is the fact that in upwards of 400 cases of recovery (herein are included those observed by M. Louis since the publication of his first edition,) this observer has met with a single example only of persistence of delirium after the cessation of febrile reaction; a fact, he well observes, incompatible with, and in itself sufficient to disprove the correctness of the notion that the delirium in this disease depends upon appreciable cerebral lesion.

How stands the question with regard to the relation of gastric changes and delirium? Let us display M. Louis' facts, bearing upon this point also, tabularly:

| Mucous membrane of the stomach. | 12 subjects Non-delirious, or so for last two or three days of life. | 12 subjects More or less violently delirious. |
|---|---|---|
| Healthy except from slight pink tint . . . | . . . . . 1 | Natural . . . 2 |
| Slightly softened at great cul-de-sac or pylorus . | . . . . . 3 | . . . . . . . 2 |
| Softened and attenuated . | . . . . . . . | . . . . . . 2 |
| Seat of a few small ulcerations . . . . | . . . . . 2 | |
| Mammillated, and commonly changed in colour, consistence, and thickness | . . . . . 6 | . . . . . . 6 |

On the independence of the two conditions under consideration here displayed it is unnecessary to insist; but it is important to add that neither the stomach *nor* the brain presented any remarkable change in several cases. The general inference drawn by M. Louis as to the causation of the delirium is that "as one lesion only is of constant occurrence and of similar character in all these subjects—namely that of Peyer's glands—in that lesion and doubtless in the agencies, whatever they may have been, favouring the development of the affection, resides the cause of delirium, and more especially of somnolence." But that the febrile action present is the intermediate and more direct cause appears evident from the extremely general occurrence of such action in the subjects of all acute diseases, whereof it constituted the only common phenomenon.

The spasmodic symptoms, observed in patients labouring under the typhoid affection, were of two kinds, muscular stiffness and alternate muscular contraction and relaxation. To the latter class are referrible subsultus tendinum, hiccup, &c. The vastly greater frequency of symptoms of this order in fatal cases than in those of recovery shows that their occurrence materially increases the unfavorable tendency of the prognosis; and as no single instance of persistent contraction of the muscles of the neck and arms was observed in cases of recovery, this symptom may be regarded as of almost fatal augury.

In commenting upon the prostration of strength attending typhoid fever, in the majority of cases from the outset (ten out of nineteen subjects were obliged to give up their ordinary occupations on the day of attack), M. Louis carefully points out the exceptions occasionally observed. Thus an individual who died on the 24th day, continued to work during the fifteen first, and was able to get out of bed without help on the day of his death.

Out of 700 cases of acute disease of which 130 terminated fatally, 8 only were attended with œdema of the lower extremities: 6 of these were cases of pneumonia or intense pulmonary catarrh, 1 of pericarditis, 1 of scarlatina. Hence it follows that the occurrence of œdema in a subject affected with acute disease should direct the attention of the physician to the lungs or heart, provided there be no other symptom of Bright's disease present.

Redness of the conjunctiva, observed in 16 of 27 fatal cases, was the produce either of uniform discoloration or of injection. Almost every individual suffered from slight intolerance of light in the sitting posture. Total spasmodic occlusion occurred towards the close in 4 fatal cases.

Extreme deafness does not increase, as has sometimes been stated, the probability of a fatal issue. Suppuration of the auditory canal occurred in a few instances; a state which has been observed to terminate in perforation of the tympanum.

The eruption, termed *pink lenticular*, consists of minute spots, variable in number, of the shape and tint indicated by their name, slightly papular or elevated above the surrounding surface, disappearing completely under, and reappearing on the removal of, pressure—characters all of them distinguishing these maculæ from true petechiæ. The frequently late arrival of patients in hospital prevents the earliest possible period of their development from being easily discovered. The mean time of their appearance has already been estimated approximatively. It is remarkable that the frequency of their occurrence and their abundance are not in the direct ratio of the severity of the disease. Desquamation of the epidermis, attended or not with development of sudamina, was observed in some cases.

The statement of the conditions of the pulse shows, as might have been anticipated, that the absence of great rapidity is a favorable sign. In respect of relation between the states of the pulse and of the tissues of the heart, M. Louis found that "of 41 subjects whose pulse was properly examined, 13 suffered from softening of that organ, and almost all of them to a remarkable extent. On the other hand, 11 of 17 patients in whom the pulse was unequal, irregular, intermittent, small, weak, tremulous, and contracted, for a shorter or longer period, presented the same lesion on post-mortem examination; . . . . . this organ was consequently in the natural state in $\frac{2}{3}$ only of the cases in which the pulse possessed the characters referred to." This induction gives additional verisimilitude to the inference already drawn respecting the period at which such softening originates; while it points out a means of ascertaining that period during life with an approximation to certainty.

The voice was changed in character, or suppressed, in cases attended with the development of pseudo-membrane in the air-passages; M. Louis has since the publication of his first edition met with an instance wherein complete aphonia persisted upwards of thirty days, and was probably dependent on ulceration of the larynx.

Some circumstances give diagnostic value to the ronchus referred to under the head cough: its universality and loudness distinguish it from that of acute primary bronchitis; and a special characteristic arises from the want of agreement between its extent and violence and the amount of accompanying dyspnœa.

The state of the blood in the veins after death did not always correspond with that observed during life; fibrinous clots were occasionally discovered in subjects whose blood furnished only a soft gelatiniform clot, when abstracted from a vein. From this M. Louis is inclined to infer that the morbid states of the blood, as those of the viscera, pursue a retrograde course after a certain period of the disease. He refers briefly to the recent researches of MM. Andral and Gavarret,* which, while they prove the fact of diminution in the ratio of fibrine in the blood to be greater in typhoid fever than that observed in any other disease, and directly proportional to the severity of the malady, also show that the same species of diminution arises in eruptive and simple continued fevers, in congestions and in cerebral hemorrhage. The globules are found, especially if the enquiry be undertaken at any early period of the affection, far from having diminished, to have notably increased in proportional quantity.

*Diagnosis.* In the chapter on diagnosis, to which we have now conducted the reader, are considered the grounds for affirming the existence of this particular disease in severe and slight cases, the peculiarities of *latent* and *simulated* typhoid fever, the question of the identity or non-identity of the disease with Camp typhus and the typhus fever of these islands.

Our examination of this chapter will be appropriately introduced by a transcript of M. Louis' excellent definition or rather condensed description of the disease:

"The typhoid affection may be characterized as an acute disease, attended with febrile action of variable intensity; of uncertain duration; proper to young subjects, more especially to those recently exposed to external conditions, to which they had previously been unused; commencing with violent rigors, anorexia, thirst, and in the very great majority of cases with diarrhœa; attended at an early period with a degree of weakness more than commensurate with the severity of the other symptoms, next with somnolence, giddiness, disturbed vision, stupor, delirium, meteorism, enlargement of the spleen, the formation of sudamina, of peculiar lenticular pink spots, of eschars over the sacrum, of ulcerations in the situations where blisters may have been applied; with deafness, various spasmodic motions or persistent contraction; occasionally with intestinal hemorrhage; very rarely with aphonia: a series of symptoms which progressively increase in intensity, when the disease is destined to terminate

* Vide Brit. and For. Med. Rev., vol. XI. p. 242. Jan. 1841.

fatally, and diminish more or less rapidly until they have totally disappeared if the contrary be the result: the whole group being characterized anatomically by a special morbid change of the glandular patches of the ileum." (Vol. ii. p. 195.)

With the exposition of the number and mode of catenation of these symptoms warranting the observer in diagnosticating typhoid fever we cannot tarry, but the minute study of these paragraphs is indispensable, were it only to demonstrate the occasionally almost insurmountable difficulty of the diagnosis. We find that so late as 1830 M. Louis himself, the greater part of whose medical life has been devoted to minute investigation of the malady, mistook symptoms originating in central softening of the brain for those of the malady in question. And yet this is the affection respecting the diagnosis of which the mere dicta of practitioners, who have never demonstrated directly or indirectly their capabilities for the task, are unhesitatingly accepted as correct,—the word of clinical tyros even deemed a sufficient guarantee,—nay more the random assertion of hospital patients of the lowest grade registered without comment in lieu of more worthy evidence, and hence virtually made to establish the existence or non-existence of "typhus" in a relative, a family, the inhabitants of a street, or the population of a district!* From the case referred to it follows that an affirmation of the existence of typhoid fever is not perfectly warrantable even "where there is considerable fever, accompanied with intense cephalalgia, great prostration of strength, dryness and brown discolorations of the tongue, *if no abdominal symptom be present;* . . . . . to authorize the physician in such diagnosis beyond all fear of error there should be present in addition a certain amount of meteorism, a few lenticular spots, disturbance of the functions of the organs of sense, enlargement of the spleen, and nasal hemorrhage."

M. Louis establishes an elaborate parallel between enteritis and the typhoid affection; diseases which the specious fallacies of Broussais had succeeded in confounding. Made acquainted with the error of these by the publication of our author's first edition, his countrymen now without an exception recognize the essential dissimilarity of the two maladies. In England, however, we have rarely known typhoid fever become the topic of conversation, without hearing it affirmed that M. Louis considers the disease an enteritis or a gastro-enteritis. Much has already appeared in the foregoing pages subversive of this error, but as the existence of correct notions on this point is of essential consequence, we transcribe M. Louis' tabular view of the symptoms in a number of cases of each of the affections in question. The cases of typhoid fever belong to a different series from those analyzed in the work.

* It is a notorious fact that in these islands the question of the contagious origin of the disease has in multitudes of instances been determined, not from personal examination, by the physician or by competent delegates, of the individuals alleged to have communicated the disease, but by the mere circumstance of such allegation having been made. Upon this point we shall have occasion to speak further in a second article, when considering the nature of the evidence existing upon numerous questions connected with the typhoid fever of this country.

| TYPHOID AFFECTION. 17 cases of recovery. | ENTERITIS. 23 cases of recovery. |
|---|---|
| **1. *Diarrhœa.*** | |
| In 14 out of 15 cases: from 6 to 10 stools in 5 cases, less in the others. | In 23 out of 23 cases: from 7 to 30 stools in 21 cases, less in the others. |
| **2. *Abdominal Pain.*** | |
| In 8 out of 11 cases. | In 22 out of 23 cases. |
| **3. *Meteorism.*** | |
| In 12 out of 17 cases. | In 1 out of 23 cases. |
| **4. *Spleen.**** | |
| Enlarged in 11 out of 15 cases. | Not enlarged in any. |
| **5. *Epigastrium.*** | |
| Somewhat painful during cough in 1 case. | Not painful. |
| **6. *Nausea.*** | |
| In 1 case. | In 2 cases. |
| **7. *Appetite.*** | |
| Null in every case, until convalescence. | Null in 5 out of 23 cases on the admission of the patients; rapidly recovered. |
| **8. *Tongue.*** | |
| Thickened and red in 3 cases; dry and red in 2; dry and black in 1. | Sometimes whitish, without other change. |
| **9. *Fauces.*** | |
| Inflammation of the velum and uvula in 2 cases; of the pharynx and tonsils in 1. | Natural. |
| **10. *Cephalalgia.*** | |
| In every case from the outset and often intense. | Slight in a single case. |
| **11. *Somnolence.*** | |
| Tolerably marked in 5 cases. | Null. |
| **12. *Intelligence.*** | |
| More or less disordered in 6 cases. | Intermittent delirium (removed by bark) in 1 case. |
| **13. *Strength.*** | |
| Extreme weakness in 7 cases. | No prostration of strength. |
| **14. *Dazzling before Eyes.*** | |
| In the only 6 cases in which mentioned. | None. |
| **15. *Spasms.*** | |
| Of the lips in 11 cases, without subsultus. | None. |
| **16. *Tinnitus Aurium.*** | |
| In 8 cases. | None. |
| **17. *Deafness.*** | |
| In 3 cases. | None. |
| **18. *Vision.*** | |
| Disturbed in 7 cases. | Unimpaired. |
| **19. *Epistaxis.*** | |
| In $\frac{7}{10}$ of the cases. | None. |
| **20. *Pink lenticular Maculæ.*** | |
| In 15 out of 16 cases. | None. |
| **21. *Sudamina.*** | |
| In 9 out of 12 cases. | In 1 case of dysentery complicated with pneumonia. |

* The fact of such enlargement is to be roughly ascertained by the freedom from or presence of fulness and tension in the left hypochondrium; the increase of size to be measured with precision by percussion.

| TYPHOID AFFECTION. | | ENTERITIS. |
|---|---|---|
| | 22. *Rigors.* | |
| In 11 out of 12 cases. | | In 5 out of 23. |
| | 23. *Heat of Skin.* | |
| Unnaturally great in every case. | | Slightly so in 4 cases of dysentery. |
| | 24. *Sweats.* | |
| Copious in 5 cases. | | In 9 cases, the only ones in which the symptom mentioned. |
| | 25. *Pulse.* | |
| Above 100 in 7 cases. | | 80 in 3 cases for one day; between 50 and 70 in the others. |
| | 26. *Duration.* | |
| Mean = 25 days in the case of patients admitted from the 6th to the 9th day; = 30 in those admitted after that period. | | Mean = 3 or 4 days counting from the admission of the patients; they left after a sojourn of 8 days. |
| | 27. *Mortality.* | |
| Besides the 17 cases under consideration 4 fatal ones occurred within the same period, which gives a mortality of 1 in 5. | | None. |
| | 28. *Age.* | |
| Mean = 23·5<br>Extremes = 13 and 35. | | Mean = 36.<br>Extremes = 15 and 70. |

*Modifications of form of typhoid fever.* The author next proceeds to the consideration of those categories of cases, the existence of which appears on first sight to shake materially the foundation on which his doctrine is erected. The first of these comprehends five cases, to which the title of "latent typhoid" is given, wherein death arose from perforation of the ileum, previously to which occurrence the symptoms had been of such character as if not to exclude, at least not to suggest, the idea of their dependence upon typhoid fever. The second refers to four cases characterized by intensely marked symptoms, while the Peyerian lesion was deficient on first view if not in specific character, at least in the development which was fairly to be anticipated. In a third class are reported three cases in which the majority of the symptoms of the typhoid affection occurred without specific change in the patches of Peyer. The apparent objection derivable from the first order of facts against the constant coexistence of the group of symptoms and anatomical change described in these volumes, is met by the consideration that a similar latent course occasionally masks the symptoms of the severest acute and chronic diseases,—that pneumonia even would frequently escape detection were the functional phenomena alone trusted to in the attempt to discover its existence, and that individuals sometimes perish from tuberculous perforation of the pleura in whom the ordinary symptoms of phthisis had never developed themselves. Now from cases like these the deduction would not be that there is no particular series of symptoms referrible to pneumonia and to phthisis, but simply that the ascertained effects of these maladies are occasionally not evolved. The objection which may be urged on the score of the second set of cases is open to similar refutation: cases are on record, and we have ourselves seen such, where pneumonia has been attended with the severest symptoms and eventually terminated fatally, and yet a *small* portion of a *single* lung only been found affected with the *second* degree of inflammation. Such instances might easily be multiplied. It has been objected to M. Louis, in respect of the third class of cases that in refusing these the title of typhoid fever, because the patches of Peyer were healthy, he falls into a *petitio principii.* It is argued by M. Dalmas, "you propose to enquire if the group of symptoms termed typhoid fever is invariably attended by a given lesion, and when the lesion which occurs most frequently happens to be absent, instead of drawing the conclusion that such lesion is not of constant occurrence, you infer that the case was not one of typhoid fever,—because the lesion, which you call characteristic, was absent. But the precise question is whether that lesion is or is not characteristic." This argument is indubitably a strong one, but *audi alteram partem.* Analogous circumstances arise, it is urged, in the instance of diseases the anatomical character of which is thoroughly and definitively established by general experience; the only fair version of the difficulty attending these cases of "simulated typhoid fever" is that M. Louis was deceived by appearances, as others have been in respect of the affections just alluded to. "The symptoms of croup," says M. Valleix,* "have been supposed in certain instances to be present by most skilful practitioners—tracheotomy even been on the point of being performed for their relief, when death has cut off the patient and not a particle of false membrane appeared in the larynx. What is the conclusion? That croup occurs without false membrane? Certainly not, but that the symptoms of croup were *simulated* by those of spasmodic stridulous laryngitis,—that the case in short was one of pseudo-croup, and the diagnosis erroneous. Again, meningitis has often been presumed to exist, in cases wherein the dissection disclosed none of its anatomical characters. Nay, cases are cited in which all the rational, and even the physical signs of vesical calculus, among others the shock of the sound against a resisting body having been manifestly present, the patient has been cut and no stone discovered."† What was the inference here, but that men of the first powers are under certain circumstances liable to be deceived. And this style of reasoning derives support from the fact that within the last ten years, a period during which the diagnosis of the malady may be considered to have attained extreme perfection, not any, or scarcely any, exceptional cases have been encountered.—For our own parts, during an experience of four years in the Parisian hospitals, we never saw a single instance of the symptoms occurring without the lesion, or of the development of the latter unattended by the former;—but the Peyerian change was in more than one case so slightly marked, as to be barely capable of identification. But more of this hereafter.

M. Louis enquires into the similarity or dissimilarity of Camp typhus and typhoid fever, and deriving his knowledge more especially from the Memoir on the subject by M. Gauthier de Claubry, coincides with him in considering the symptoms of the former affection, as far as the vague language of its describers warrants the formation of an opinion, to be those of typhoid fever. The justness of this conclusion is considered incontestable, in respect of the occurrence of a certain number of symp-

* Archives Générales de Médecine, Janvier, 1839.

† We remember to have heard M. Roux mention many years since his having been five or six times unfortunate enough to lithotomize non-calculous patients.

toms—diarrhœa, abdominal pain, meteorism, lenticular maculæ, occasionally petechiæ, nasal hemorrhage, deafness, disturbed vision, somnolence, stupor, and delirium. In both diseases the course of the disease may be rapid or slow; the mean age of their victims is very nearly the same, the occurrence of typhus after the fiftieth year being apparently very rare; while both diseases, with extremely rare exceptions, affect the same individual once only. Where the viscera have been examined with any degree of closeness, the anatomical changes appear to be those observed in the typhoid affection,—gangrenous eschars, ulcerations of the mucous membrane of the small intestine, tumefaction, softening, and gray discoloration of the mesenteric glands corresponding to the seat of the intestinal ulcerations. These data appear to M. Louis sufficient to warrant an affirmation of the "identity" of the two diseases. Neither the silence of the describers of "typhus," respecting perforation of the intestine—an occurrence far from uncommon in the course of typhoid fever—nor the alleged fact that the symptoms of "typhus" have of late years been observed at Rochefort and Toulon without the existence of the intestinal lesion being established, appear to our author to constitute sufficient motives for renouncing or altering the opinion just expressed. The latter circumstance appears to him simply to indicate "the simultaneous existence of two severe diseases,"—an inference into the justness of which we shall have occasion hereafter to enquire, when we examine the section of these volumes referring to the typhus of our own country.

Perforation of the small intestine proved the immediate cause of death in 8 out of 55 fatal cases. Commonly single, the perforation was in some instances double or triple, seated in the vicinity of the cæcum in the midst of the ulcerated patches. The most remarkable circumstance perhaps connected with it was its occurrence in the majority of instances in mild cases; to such an extent that five of these belonged to the class *latent.* Perforation took place on the 12th day of the disease in one case, the 18th in two, from the 22d to the 42d in the remaining five. In five cases the *sudden* supervention of the usual symptoms of intense peritonitis announced the accident; in three it followed almost a latent course, though proving as promptly fatal as in the others. In certain instances the first severity of the abdominal pain may be mitigated; but the persistence of retraction of the features, of nausea, or vomiting, and of rigors show that the real gravity of the case has in no wise diminished. In some subjects the prevalence of cerebral symptoms notably masks those of perforation. The period elapsing between the occurrence of the accident and death varied, except in the instance of one subject, between 20 and 54 hours; the individual in question survived until the seventh day.

*Prognosis.* M. Louis' chapter on prognosis does no more than exhibit in a condensed form the facts scattered through his previous pages, whereby the almost impossibility of forming an assured judgment as to the issue of the malady is rendered very painfully evident. The relative prognostic value of particular symptoms may be gathered from the subjoined table, which we have drawn up from our author's descriptions; from this it appears that those enumerated under the head D, are the only ones at all warranting a fatal prognosis, and even these appear to arise—in infinitely rare cases, it is true—in individuals destined to recover.

The fact just referred to respecting the more usual occurrence of perforation in mild than in severe cases, displays the complexity of the question of individual prognosis in another aspect.

| A. *Symptoms which occur with the same or almost the same frequency and intensity in fatal cases and in those of recovery.* | B. *Symptoms which occur with greater frequency, but not to any notable degree, in fatal cases.* | C. *Symptoms occurring with much greater frequency in fatal cases.* | D. *Symptoms peculiar to, or with the rarest exceptions peculiar to, fatal cases.* |
|---|---|---|---|
| Diarrhœa.<br>Gastric symptoms.<br>Morbid states of the tongue.<br>Prostration of strength.<br>Epistaxis.<br>Deafness.<br>Tinnitus aurium.<br>Sudamina.<br>Lenticular eruption. | Involuntary dejections.<br>Meteorism.<br>Dysphagia.<br>Ocular injection.<br>Marked rapidity of pulse.<br>Irregularity or intermittence of the pulse. | Intestinal hemorrhage.<br>Delirium *appearing the first day.*<br>Agitation.<br>Somnolence.<br>Spasmodic movements.<br>Extreme prostration of strength from the first.<br>Permanent disturbance of vision in the recumbent position.<br>Erysipelas.<br>Persistence of a small contracted pulse. | Somnolence appearing the *first* day.<br>Perversion of intelligence exhibited by patient declaring himself well, while in reality suffering under very severe symptoms.<br>Persistent muscular contraction. |

There is one circumstance which, according to M. Chomel's experience, may justify a positive opinion as to the fatal issue of the malady, namely, severe relapse after short remission of the symptoms. Slow invasion of the disease also augurs ill for its mode of termination. Certain circumstances connected with the general condition of the individual modify the prognosis; of these the most important is age. The younger the subject, provided he be an adult or nearly so, the stronger his chance of recovery; M. Louis has in the course of the last ten years seen only one person aged less than twenty succumb. From a very admirable recapitulation made by M. Chomel in 1835, having for its basis the cases observed in his wards at the Hôtel-Dieu for three years, and of which we have the numerical results now before us, it follows that the prognosis is most favorable in the case of individuals under twenty years of age, of the female sex, attacked during the summer and resident at Paris more than two years.

*Etiology.* Passing to the chapter on etiology, we find M. Louis displaying the ages of his patients as follows:

| Ages. | Fatal cases. | | Recoveries. |
|---|---|---|---|
| 17 to 20 | 14 | (15½-20) | 31 |
| 20 — 25 | 20 | | 39 |
| 25 — 30 | 11 | | 13 |
| 30 — 39 | 5 (=50) | | 5 (= 88) |

The mean age in the fatal cases was 23, in those of recovery 21; the favorable chances afforded by youthfulness appear more distinctly from considering the much greater proportion of recoveries than of deaths before the twenty-fifth year.

LIBRARY OF THE

Orleans Parish Medical Society

Of 138 typhoid subjects, 32 only were females. M. Louis, however, correctly considers this result not to be confided in as the expression of a general law, inasmuch as the number of males constantly arriving in Paris, and unable to remain at home during illness, is probably much greater than of females,—a notion confirmed by facts observed in some rural districts of France. The mortality in M. Louis' cases was equal in both sexes, wherein his experience differs from that of M. Chomel.

The division of typhoid individuals into two classes in respect of trades, those requiring considerable expenditure of strength and the contrary, shows these conditions to be unimportant in respect of the development of the disease.

| | Laborious trades. | Non-laborious trades. |
|---|---|---|
| No. of cases | 77 | 55 |
| Deaths | 28 | 18 |

And the unimportance of the trifling excess in the ratio, furnished by individuals following laborious occupations, is manifest from the fact that in all probability the *laborious* are the best-peopled trades, if we may so speak, in Paris.

From the facts observed by M. Louis it would follow that strength or feebleness of constitution, excessive manual labour, moral suffering, and over indulgence in alcoholic beverages, do not distinctly act as causes of the malady. "Nor can a sojourn during the night in cellars inhabited by an excessive number of persons be esteemed a cause of the disease; the eighteenth part only of the subjects observed having been sufferers from such deficiency of healthful lodgement."

Removal from the provinces to Paris favours the outbreak of the disease; but the longer the stay of the patient in that town, the greater, as already mentioned, are his chances of recovery. This is shown in the following table:

| Length of residence in Paris. | Deaths. | Recoveries. |
|---|---|---|
| 2 to 3 weeks . . . | 10 | |
| 2 weeks to 3 months . . | ... | 7 |
| 3 months to 5 months . . | 8 | 19 |
| 6 10 . . | 10 | 19 |
| 11 20 . . | 9 | 20 |
| 20 30 . . | 5 | 12 |
| 30 40 . . | ... | 1 |
| 3 years to 8 years . . | 2 | 7 |
| | 44 | 85 |

The mortality among those who had been in Paris a less period than ten months was 38·36 per cent.; among those whose residence had been longer, 28·6 per cent. The proportions expressed in the table, though approximations to the truth, cannot, as M. Louis himself well observes, be considered possessed of perfect precision; their claim to this character is based on the presumption, "that all the working classes in Paris, of all ages, seek hospital aid in the same proportion;" now this is not exactly the truth, as a tolerably large share of those who have inhabited Paris for years remain at their own homes. The question naturally arises, what is the cause of this predilection of the disease for the class of persons referred to? We have just seen that misery of circumstances cannot have had any material influence on the production of the disease; and the bearing of the fact already noticed coincides with that of another observation by M. Louis, namely, that "the majority of the patients were of good constitution, their flesh was firm, their complexion of the ordinary kind, and they possessed a fair share of fatness when they were admitted into hospital; they were consequently in a condition subversive of the notion of wasting away, or of the prolonged action of some deleterious agency, capable of influencing the function of nutrition." It is true, nevertheless, observes our author, that practitioners commonly ascribe very powerful influence to bad nourishment in the production of typhoid fever; but while he recognizes, as all must, the reality of the pernicious influence of such nourishment on health, and in the development of disease in general, he finds himself obliged to declare that he has looked in vain for "incontestible proof that such influence is more marked in respect of typhoid fever than of any other disease, even of that typhoid fever which follows in the train of armies, under circumstances wherein a multitude of sources of insalubrity coexist,—atmospheric exposure, privation of food, excesses of every kind, and, as regards the sick and wounded, accumulation of a number of persons in the same place." The well-known experiments of M. Gaspard are correctly viewed by M. Louis as proving nothing more than that the injection of putrid matter into the veins of animals will give rise to a series of severe symptoms frequently assuming a typhoid character, (as may be the case with almost every acute disease to which the frame is liable), while the lesions produced do not wear the characters of those of typhoid fever, and seem to originate in almost all organs indiscriminately. Besides, it is perfectly illogical to compare the agency of a cause acting violently and suddenly with one producing its effects gently and slowly. Had M. Gaspard shown that animals fed for a length of time upon putrid substances became eventually the subjects of symptoms and lesions analogous to those of typhoid fever, his experiments would have assumed a very different share of importance.

CHARITY HOSPITAL, MEDICAL LIBRARY

The archives of the Medical Society of Observation contain the record of a case observed at the Hôpital St. Louis by M. Letenneur, which *apparently* supplies an argument of much sounder character and higher pretensions in the direction under consideration. An individual aged 21, of good constitution, having inhabited Paris for fifteen months, of sober habits, habitually using nutritious food, occupying a well ventilated room on the fourth story, and working by day in a spacious workshop with a southern aspect, ordinarily enjoying excellent health, but for the last sixteen months suffering from obstinate blennorrhœa, which had resisted all the usual modes of treatment, was admitted into hospital with the symptoms of an acute affection. Upon enquiry it appeared, that he had for some time been in the habit of drinking twice a day a glass of water in which mashed straw had been macerated for ten or eleven days, (a popular remedy in Paris for gleet); and, according to his fellow-workmen, had consumed altogether four litres (about five quarts) of the liquid. When the first symptoms of his existing malady declared themselves, the urethral discharge had then almost disappeared. The smell of the liquid was so horrible and so tenacious, that repeated washing failed to remove it from the vessel used for maceration. The patient had been five days ill on that of his admission: the case followed the course of typhoid fever; and after death, which occurred on the tenth day, the characteristic lesion of the patches of Peyer exhibited itself: all these points are clearly shown in the full narrative of the case, from which we have merely extracted the more striking particulars. But, argues M. Louis, this case is more apparently than really conclusive: it is a solitary one, and the development of typhoid fever may have been a mere coincidence, especially as individuals constantly enjoying most nutritious diet are not exempt from the disease. Besides, if the ingestion of putrid water were often attended with consequences such as those some persons might in the present instance feel disposed to attribute to it, the remedy would decidedly be less cherished than it is by the lower orders in Paris. And in support of M. Louis' arguments may be adduced the facts, that quantities of putrescent fish are habitually consumed in some of the northern islands, and that the Esquimaux subsist in great measure upon rancid oil, without being on this account, if the narration of travellers may be credited, particularly subject to the ravages of typhoid fever. It is further urged by M. Louis that, while the disease is not observed to occur after the age of fifty, it is precisely among workmen who have passed that age that the greatest difficulty in earning sufficient and healthful nutriment prevails.

*Contagion.* One of the novelties enriching the present edition of M. Louis' volumes is a chapter on the subject of contagion. Of the reality of contagion as a cause of the propagation of typhoid fever the following facts, among many of similar character, afford in M. Louis' opinion full demonstration. M. Bretonneau, of Tours, has seen the disease pass from one village into another previously free from it, after the arrival in the latter of a person who had sojourned for a more or less long period in the former. M. Gendron observed, that after the arrival of a diseased individual in a village, the malady spread from house to house, when communication had occurred between the inhabitants of these; and that the persons most invariably and earliest seized were those who had been most assiduous in their attentions to individuals suffering under the malady. Similar facts have been observed in country hamlets by M. Putegnat. In all these cases the lesion of the patches of Peyer was invariably discovered when an autopsy took place. Respecting the contagiousness of the disease, exemplified by such occurrences as these, M. Louis does not think warrantable doubt can be raised, so far as rural districts are concerned. If admitted to prevail in these, it would seem an *à priori* necessity to recognize its existence in towns. The Parisian physicians, wisely rejecting on general principles such mode of reasoning, believed themselves justified by an appeal to direct observation in denying the reality of contagiousness in the capital they inhabit. It has been urged by M. Andral, that the disease is not observed to be transmitted in hospitals from a typhoid patient to those occupying the adjacent beds. And again, circumstances most favorable to the action of contagion appear to exist in the instance of medical students who closely tend their typhoid companions in small and ill-ventilated rooms, such as they commonly inhabit; yet the same observer has never met with a single instance of its development under these conditions. Against the views, apparently derivable from these facts, appears, in the first place, the general argument stated by M. Bretonneau, that it must be extremely difficult from the very nature of things to trace the movement of contagion in populous towns. In respect of M. Andral's objections it is urged by M. Louis that the transmission of indubitably contagious maladies, such as measles, is scarcely ever observed in hospitals for adults; and if the validity of this argument be demurred to on the ground of measles being a disease of infancy, the demurrer must be entered in the case of typhoid fever also, for this too occurs in a very large proportion of cases at a similarly early period of life. Besides the fact of measles being a more contagious disease than typhoid fever is, according to M. Louis, questioned by none. Further, our author has three times observed the development of the disease in his own wards; two of these cases occurred during an epidemic. The argument drawn from the safety of students filling the office of *garde-malades* appears to M. Louis to lose its importance, from the fact that these youths constantly relieve each other in their office of friendship, and hence are not continuously exposed to the chances of contagion for any lengthened period. The large share of artisans and students who are seized with typhoid fever within a limited period after their arrival in Paris, argues in favour of its contagious nature. Admitting, in truth, the reality of contagion, it is easily intelligible that youths arriving in a town where the disease constantly exists, should contract it in greater or less number through direct or indirect communication with affected individuals. It may be objected to the notion of the contagionists, that typhoid fever differs from confessedly contagious disorders, in that the anatomical change characterizing it, originates in the great majority of cases with the first symptoms. The premonitory symptoms of the exanthemata are here referred to; M. Louis exposes the trivial character of the objection. There are some other interesting points in this chapter discussed or referred to, of which, as well as of the whole line of argument here adopted, we reserve our examination for a future occasion. To that period we also postpone the consideration of M. Louis' therapeutical results.

The volumes, of which we for the present take leave, are appropriately dedicated to the members of the Medical Society of Observation. Few, indeed, could urge a stronger claim for such distinction than they who have exhibited unwearied zeal in following out the system of their illustrious president, and accumulating additional demonstration of the value of that system by applying it to previously unexplored branches of enquiry. But if M. Louis be indebted to the ardour of his disciples, the benefit he has conferred upon them, and indeed upon all observers who may be charged with "the atrocious crime of being *young men*," is of even a higher order. He has established and promulgated the validity of a method of studying disease, which invests the young with equally great power to extend the limits of their science as the old, provided they approach their task with steadfastness, energy, and honesty, without which none can hope to secure even a single pebble on the shore of that great ocean—truth. We need only place before the reader the names of Valleix, Bizot, Barth, Grisolle, Rufz, Gerhard, Jackson, Maunoir, Woillez, Rilliet, Taupin, Nélaton, and D'Espine, to remind him of the extent and valuable character of services which youth, guided in its labours by minute observation and the numerical method, may render to positive medicine. And there is a related good accomplished, as well for its cultivators as for the science itself, by M. Louis, the true bearing and importance of which do not appear to us to have been correctly understood or estimated. He has perfected an engine whereby *authority* may be resisted. He has unmasked that curious despotism (assumed by certain individuals in consequence commonly of their worldly position, or conferred on them by others who hoped thereby in turn to wield it,) which had been enabled to crush the efforts of young and ardent searchers after truth, did the results of these efforts—no matter by what diligence of observation and closeness of induction they were obtained, no matter how clearly the style of the narrative demonstrated the capability and conscientious exactitude of the observer—clash in the remotest degree with the dogmata it pleased themselves to originate or foster.* While the system of *authority* prevailed there was but one mode of reception for the results of *unpatronized* labour:

> "Tous ces discours sont des sottises,
> Venant d'un homme sans crédit;
> Ce seraient paroles exquises,
> Si c'était un grand qui les dit."

Had M. Louis done nothing more than thoroughly exposed the hollowness of all evidence founded on the authority of name, while he raised to its proper level that of facts, the single achievement would have ensured him an imperishable name among the benefactors of medical science. He has thereby, in truth, destroyed an ever active organ of evil, "the greatest and most irreconcileable enemy to truth and argument," (to use the energetic language of Bishop Hoadley, with a still higher topic for his theme,) "that this world ever furnished forth. All the sophistry, all the colour of plausibility, all the argument and cunning of the subtlest disputer in the world, may be laid open and turned to the advantage of that very truth which they designed to hide or to depress, but against *authority* there is no defence. It was authority which would have prevented all reformation where it is, and which has put a barrier against it where it is not."

* The spirit of unbounded respect for authority is, it would appear, only "scotched, not killed," on this side the channel. At least, we have been recently called on to notice a fact tending to exhibit the vitality of the mischief in a manner painful to all conscientious lovers of science. A learned and most amiable reviewer at the commencement of an article upon the recent work of certainly a very eminent and excellent man, but still a man, not only disclaims all intention to enquire into the correctness of a solitary one of the multitude of opinions he is about, by transcription, to aid in disseminating, but expresses his conviction that none will be found possessed of the temerity or malignity to venture upon so signal a breach of conventionalism. The temerity and malignity to labour in the cause of truth! Suppose a man distinguished for expertness as a chemist and an astronomer were, under the influence of "temporary mental derangement," to assert the moon to be made of cheese, the very first frosty night our philosophical reviewer of the *authority* school would, doubtless, distinguish the rind, smell the caseous odour, and clearly perceive parties of mites taking an airing on its surface, and probably exert his critical powers to determine whether the product of celestial churning belonged to the Stilton, the Chedder, or the Gloucester varieties.

